# Prognosis of mechanically ventilated patients with COVID-19 after failure of high-flow nasal cannula: a retrospective cohort study

**DOI:** 10.1186/s12931-024-02671-y

**Published:** 2024-03-01

**Authors:** Dong-gon Hyun, Su Yeon Lee, Jee Hwan Ahn, Sang-Bum Hong, Chae-Man Lim, Younsuck Koh, Jin Won Huh

**Affiliations:** Department of Pulmonary and Critical Care Medicine, Asan Medical Center, University of Ulsan College of Medicine, 88 Olympic-ro 43-gil, Songpa-gu, Seoul, 05505 Republic of Korea

**Keywords:** COVID-19, Noninvasive ventilation, Respiratory distress syndrome, Mechanical ventilators, Critical care outcomes

## Abstract

**Background:**

There is an argument whether the delayed intubation aggravate the respiratory failure in Acute respiratory distress syndrome (ARDS) patients with coronavirus disease 2019 (COVID-19). We aimed to investigate the effect of high-flow nasal cannula (HFNC) failure before mechanical ventilation on clinical outcomes in mechanically ventilated patients with COVID-19.

**Methods:**

This retrospective cohort study included mechanically ventilated patients who were diagnosed with COVID-19 and admitted to the intensive care unit (ICU) between February 2020 and December 2021 at Asan Medical Center. The patients were divided into HFNC failure (HFNC-F) and mechanical ventilation (MV) groups according to the use of HFNC before MV. The primary outcome of this study was to compare the worst values of ventilator parameters from day 1 to day 3 after mechanical ventilation between the two groups.

**Results:**

Overall, 158 mechanically ventilated patients with COVID-19 were included in this study: 107 patients (67.7%) in the HFNC-F group and 51 (32.3%) in the MV group. The two groups had similar profiles of ventilator parameter from day 1 to day 3 after mechanical ventilation, except of dynamic compliance on day 3 (28.38 mL/cmH2O in MV vs. 30.67 mL/H2O in HFNC-F, *p* = 0.032). In addition, the HFNC-F group (5.6%) had a lower rate of ECMO at 28 days than the MV group (17.6%), even after adjustment (adjusted hazard ratio, 0.30; 95% confidence interval, 0.11–0.83; *p* = 0.045).

**Conclusions:**

Among mechanically ventilated COVID-19 patients, HFNC failure before mechanical ventilation was not associated with deterioration of respiratory failure.

**Supplementary Information:**

The online version contains supplementary material available at 10.1186/s12931-024-02671-y.

## Background

A high-flow nasal cannula (HFNC) is a noninvasive respiratory support with the clinical benefits of improving oxygenation and preventing intubation in patients with hypoxemic respiratory failure [1, 2]. In patient with coronavirus disease 2019 (COVID-19) pneumonia leading to acute respiratory distress syndrome (ARDS), oxygen therapy by HFNC has been commonly considered to avoid mechanical ventilation [3–5]. As the number of COVID-19 cases is increasing rapidly, it is known that COVID-19-related ARDS is different from other forms of ARDS [6]. Patients with COVID-19-related ARDS have two different subtypes of ARDS: type L, low elastance similar to isolated viral pneumonia, and type H, high elastance similar to classic ARDS [7]. There is an argument that patients with COVID-19 should be intubated early, because type-H patients with HFNC might undergo self-inflicted lung injury (SILI) without the clinical benefits of HFNC and type-L patients could tolerate strain without the risk of ventilator-related lung injury (VILI) [8]. In a recent clinical trial, a significant reduction in intubation rates with high-flow oxygen among patients with respiratory failure due to COVID-19 compared with standard oxygen therapy were observed, however HFNC oxygen did not reduce the mortality rate at day 28 [9]. Although it is known that delayed intubation due to failure of HFNC generally caused worse outcomes in patients with respiratory failure, the effects on COVID-19 patients remained unclear [10]. Therefore, we aimed to compare lung physiology and clinical outcomes between mechanically ventilated COVID-19 patients immediately and those after HFNC failure to investigate the effects of HFNC failure.

## Methods

### Study participants

This retrospective cohort study examined patients diagnosed with COVID-19 and admitted to the intensive care unit (ICU) between February 2020 and December 2021 at Asan Medical Center. Patients who met the following criteria were eligible for inclusion: (1) adults (age ≥ 18 years); (2) diagnosis of COVID-19 by detection of severe acute respiratory syndrome coronavirus 2 (SARS-CoV-2) nucleic acid; (3) admission to ICU for the treatment of COVID-19; and (4) patients who underwent mechanical ventilation during their ICU stay. We excluded patients who were not admitted to the ICU and those who did not receive mechanical ventilation.

### Data collection and outcomes

We collected data from electronic medical records, including demographic characteristics, medical history, laboratory results, and medications. The patients were divided into HFNC failure (HFNC-F) and mechanical ventilation (MV) groups according to the use of HFNC before mechanical ventilation. The HFNC-F group consisted of patients who initially received HFNC oxygen but finally underwent mechanical ventilation due to HFNC failure. In contrast, patients in the MV group received immediate mechanical ventilation without HFNC oxygen therapy.

Since this study’s object was to investigate whether delayed intubation after HFNC worsen lung physiology, the primary outcome of this study was to compare the worst values of mechanical ventilation parameters, including tidal volume (TV), predicted body weight (PBW), peak airway pressure, PEEP, arterial oxygen partial pressure (PaO_2_)/ fractional inspired oxygen (FiO_2_) ratio (PF ratio), and dynamic compliance, from day 1 to day 3 after the initiation of mechanical ventilation between the HFNC-F and MV groups. Dynamic compliance was calculated as TV/(peak airway pressure-PEEP). Secondary outcomes included ICU mortality, ICU discharge, and successful ventilator weaning by day 28, which were the composite outcomes of ICU discharge or ventilator weaning combined with a competing risk event and mortality within 28 days. If patients died within 28 days regardless of weaning from ventilator, these events did not contribute to the time-to-event analysis of successful weaning by setting the time to zero days [11]. We also performed the comparison of the rates of prone position, extracorporeal membrane oxygenation (ECMO), and continuous renal replacement therapy (CRRT) at 28 days between the two groups as secondary outcomes. In addition, the total duration of mechanical ventilation, length of stay in ICU and length of hospital stay was evaluated.

### Statistical analysis

Data are presented as numbers and proportions for categorical variables and means ± standard deviations (SD) or medians (interquartile range [IQR]) for continuous variables with normal distribution or non-normal distribution, respectively. The chi-squared test or Fisher’s exact test was used to compare categorical variables, while the Student’s t-test or Mann–Whitney U-test was used to compare continuous variables with a normal or non-normal distribution. For time-to-event analysis, the Kaplan– Meier method was used, whereas a log-rank test was used to test the significance of the differences. The time-to-event analysis was right-censored at 28 days. Adjustments for secondary outcomes were performed using the multivariable Cox proportional hazards regression model with covariables. We selected covariables with statistical differences for comparison between groups or *p*-values < 0.10 in the univariable analysis, considering the problem of collinearity. The proportional hazard assumption was assessed by inspecting the Schoenfeld residuals. Two-sided *p*-values < 0.05 were considered to indicate significance. All analyses were performed using SPSS version 26.0 (IBM Corporation, Armonk, NY, USA).

## Results

**Fig. 1:**
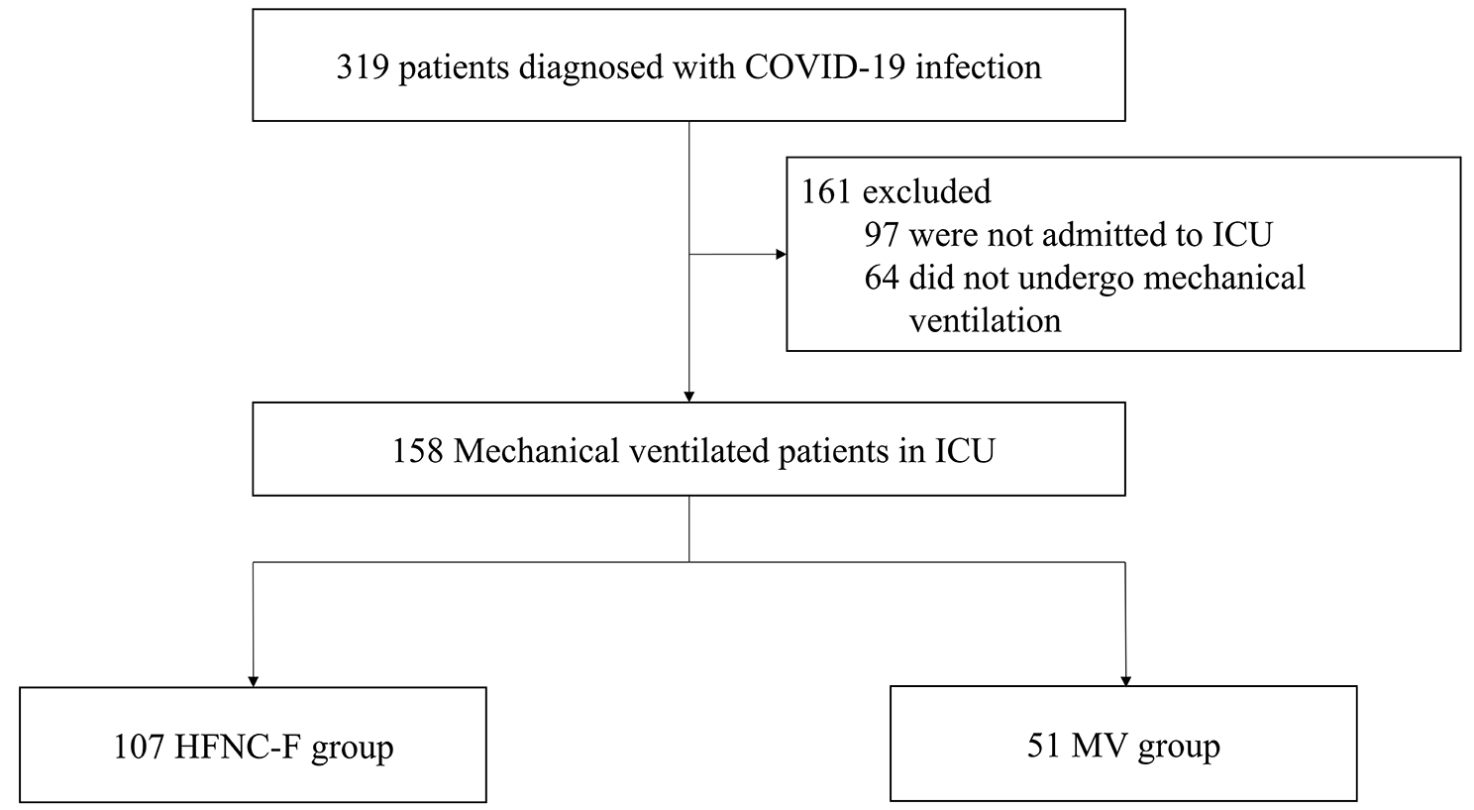
Flowchart of patients. COVID-19; coronavirus disease 2019, ICU; intensive care unit, HFNC; high-flow nasal cannula, MV; mechanical ventilation

We screened 319 eligible patients for inclusion, of whom 161 were excluded for several reasons: 97 were not admitted to the ICU and 64 did not undergo mechanical ventilation (Fig. 1). Overall, 158 mechanically ventilated patients with COVID-19 pneumonia were included in the present study: 107 patients (67.7%) in the HFNC-F group and 51 (32.3%) in the MV group.

### Baseline characteristics

The baseline demographics, including age, sex, body mass index, and comorbidities, of the two groups are shown in Table 1. There were no differences of age, sex, body mass index, and clinical frailty score between the two group, but significant differences were found between the HFNC-F and MV groups in terms of smoking status (never smoker: 60.8% in MV group vs. 74.8% in HFNC-F group, *p* = 0.008), diabetes mellitus (43.1% in MV group vs. 23.4% in HFNC-F group, *p* = 0.011), C-reactive protein level (12.3 mg/dL in MV group vs. 9.3 mg/dL in HFNC-F group, *p* = 0.020), and procalcitonin level (0.4 ng/mL in MV group vs. 0.1 ng/mL in HFNC-F group, *p* < 0.001). The profile of COVID-19 including the interval until hospital admission, status of vaccination, and treatment was similar between the two groups, except for the proportion of patients who received antithrombotic therapy (84.3% in the HFNC-F group vs. 97.2% in the MV group, *p* = 0.005). There were significant differences in pH at hospital admission (7.38 [7.31–7.44] vs. 7.46 [7.43–7.48], *p* < 0.001), PaCO_2_ (36.00 [31.10–42.70] vs. 32.00 [28.80–35.00], *p* = 0.001), and PaO_2_/FiO_2_ ratio (80.78 [53.00–154.20] vs. 110.17 [78.50–150.17], *p* = 0.047) between the HFNC-F and MV groups. The median interval of initiation of invasive ventilation from admission differed between the two groups (0.0 days in the MV group vs. 1.0 days [1.00–4.00] in the HFNC-F group, *p*-value *<* 0.001). In the HFNC-F group, the median interval from HFNC treatment to mechanical ventilation was 1.0 (1.00–3.00) days (supplemental Table 1). The median respiratory rate and respiratory rate oxygenation (ROX) index at the start of HFNC treatment was 24.0 [20.50–29.50] per minute and 4.13 [3.45–5.68], respectively. The detailed profiles according to type of respiratory support after mechanical ventilation were shown in Supplemental Table 2.

**Table 1:** Baseline demographics

Characteristic	MV (*n* = 51)	HFNC-F (*n* = 107)	*p*-value
Age, yr	65.00 [55.00, 74.00]	66.00 [58.50, 74.00]	0.553
Sex, female	20 (39.2)	50 (46.7)	0.374
Body mass index, kg/m^2^	25.00 [21.77, 29.13]	24.79 [22.66, 27.17]	0.424
Smoking status			0.008
Never smoker	31 (60.8)	80 (74.8)	
Ex-smoker	7 (13.7)	19 (17.8)	
Current smoker	13 (25.5)	8 (7.5)	
Comorbidities			
Hypertension	31 (60.8)	53 (49.5)	0.185
Diabetes mellitus	22 (43.1)	25 (23.4)	0.011
Cardiovascular disease	11 (21.6)	13 (12.1)	0.123
Chronic lung disease	5 (9.8)	7 (6.5)	0.526
Chronic neurologic disease	6 (11.8)	9 (8.4)	0.565
Chronic kidney disease	6 (11.8)	4 (3.7)	0.078
Chronic liver disease	4 (7.8)	8 (7.5)	1.000
Immunocompromised status	2 (3.9)	8 (7.5)	0.502
Transplantation status	1 (2.0)	5 (4.7)	0.665
Connective tissue disease	2 (3.9)	5 (4.7)	1.000
Hematologic malignancy	0 (0.0)	6 (5.6)	0.195
Solid cancer	3 (5.9)	7 (6.5)	1.000
Clinical frailty score	3.00 [3.00, 4.00]	3.00 [3.00, 4.00]	0.180
Profile of COVID-19			
Interval between symptom onset to hospital admission, day	7.00 [3.00, 13.00]	7.00 [6.00, 10.00]	0.893
Interval between COVID-19 diagnosis to hospital admission, day	3.00 [0.00, 12.00]	3.00 [1.00, 7.00]	0.755
Vaccination	8 (15.7)	24 (22.4)	0.324
Treatment profile			
Antithrombic therapy	43 (84.3)	104 (97.2)	0.005
Remdesivir	38 (74.5)	91 (85.0)	0.126
Dexamethasone ≥ 6 mg/day	50 (98.0)	107 (100.0)	0.323
Tocilizumab	22 (43.1)	55 (51.4)	0.331
Baricitinib	3 (5.9)	8 (7.5)	1.000
Others*	2 (3.9)	13 (12.1)	0.146
Inflammatory marker			
C-reactive protein, mg/dL	12.3 [6.7, 24.7]	9.3 [3.9, 17.4]	0.020
Procalcitonin, ng/mL	0.4 [0.2, 2.4]	0.1 [0.0, 0.3]	< 0.001
Arterial blood gas at hospital admission			
pH	7.38 [7.31, 7.44]	7.46 [7.43, 7.48]	< 0.001
PaO2, mmHg	66.80 [52.60, 97.40]	71.40 [62.00, 83.00]	0.576
PaCO2, mmHg	36.00 [31.10, 42.70]	32.00 [28.80, 35.00]	0.001
PaO2/FiO2 ratio, mmHg	80.78 [53.00, 154.20]	110.17 [78.50, 150.17]	0.047
Initiation of invasive ventilation from admission, day	0.00 [0.00, 0.00]	1.00 [1.00, 4.00]	< 0.001

Data are reported as number (percentage), median [interquartile range], or mean ± standard deviation. MV: mechanical ventilation; HFNC: high-flow nasal cannula; COVID-19: coronavirus disease 2019; PaO2: arterial oxygen partial pressure; PaCO2: arterial carbon dioxide partial pressure; FiO2: fractional inspired oxygen; SOFA: sequential organ failure assessment score

* Others included hydroxychloroquine, lopinavir/ritonavir, convalescent plasma, and immunoglobulin

### Primary outcomes

The two groups had similar profiles of ventilator parameters from day 1 to day 3 after mechanical ventilation (Table 2). The PF ratio in the first day had a mean of 92.7 ± 8.2 in the MV group and 151.3 ± 64.7 in the HFNC-F group (*p* = 0.875). There were no differences in dynamic compliance on the first day between the two groups (34.1 ± 13.4 in the MV group vs. 31.5 ± 9.4 in the HFNC-F group, *p* = 0.359). However, patients in the HFNC-F group tended to have a higher dynamic compliance on day 3 (31.9 ± 9.2) than those in the MV group (28.1 ± 10.0, *p* = 0.032).

**Table 2:** Parameters of ventilator from day 1 to day 3 after mechanical ventilation

	Day 1 (*n* = 158)		*p*-value	Day 2 (*n* = 158)		*p*-value	Day 3 (*n* = 154)		*p*-value
	MV (*n* = 51)	HFNC-F (*n* = 107)		MV (*n* = 51)	HFNC-F (*n* = 107)		MV (*n* = 47)	HFNC-F (*n* = 107)	
MV parameter									
PF ratio, mmHg	92.7 ± 8.2	151.3 ± 64.7	0.875	151.0 ± 64.0	176.2 ± 77.8	0.457	188.5 ± 83.8	169.4 ± 57.3	0.235
TV/PBW, mL/kg	6.3 ± 0.8	6.0 ± 0.7	0.446	6.3 ± 0.4	5.9 ± 0.9	0.586	6.6 ± 1.6	6.7 ± 1.4	0.533
Peak Pressure, cmH20	31.7 ± 4.3	28.5 ± 3.7	0.101	27.6 ± 3.3	25.9 ± 3.0	0.432	25.1 ± 5.2	23.8 ± 3.4	0.085
PEEP, cmH2O	14.6 ± 3.5	13.9 ± 3.2	0.881	13.3 ± 2.0	13.0 ± 1.6	0.051	10.4 ± 3.4	11.2 ± 2.4	0.118
Dynamic C, mL/cmH20	34.1 ± 13.4	31.5 ± 9.4	0.359	28.3 ± 10.8	28.4 ± 7.4	0.106	28.1 ± 10.0	31.9 ± 9.2	0.032

Data are reported as number (percentage), median [interquartile range], or mean ± standard deviation. MV: mechanical ventilation; HFNC-F: high-flow nasal cannula failure; PF ratio: arterial oxygen partial pressure/fractional inspired oxygen ratio; TV/PBW: tidal volume/predicted body weight; PEEP: positive end-expiratory pressure; C: Compliance

### Secondary outcomes

**Fig. 2:**
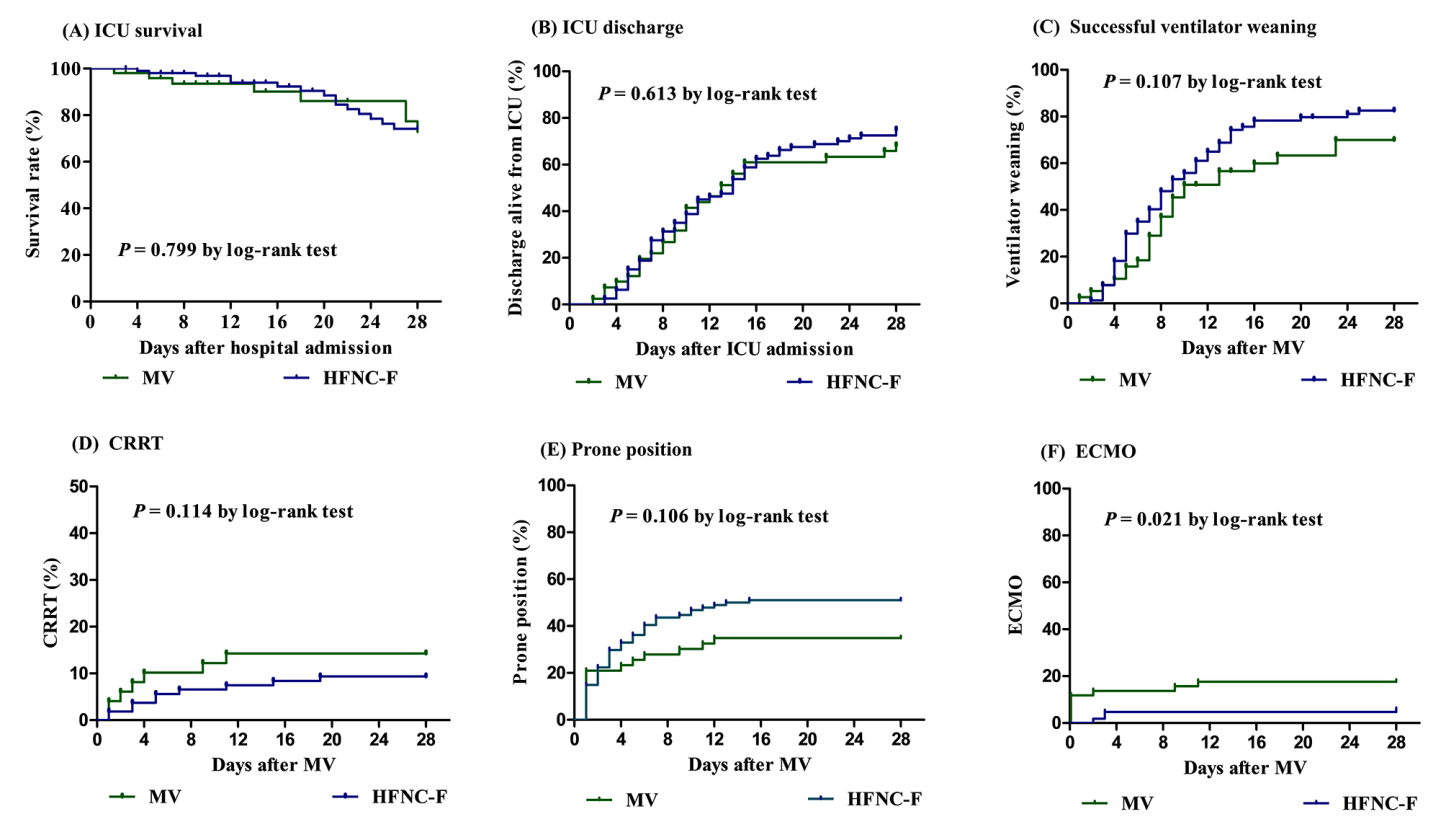
Secondary outcomes at day 28 according to the use of high-flow nasal cannula before mechanical ventilation. These Kaplan-Meier curves depict (**A**) intensive care unit (ICU) survival until day 28 as well as cumulative incidence of (**B**) ICU discharge, (**C**) successful ventilator weaning, (**D**) CRRT, (**E**) Prone position, and (**F**) Extracorporeal membrane oxygenation at day 28. MV; mechanical ventilation, HFNC-F; high-flow nasal cannula failure, ICU; intensive care unit, CRRT; continuous renal replacement therapy, ECMO; extracorporeal membrane oxygenation

The ICU mortality, ICU discharge, and ventilator weaning on day 28 did not differ significantly between the two groups (Fig. 2). Also, the two groups had similar rates of CRRT (*p* = 0.114) and prone positioning (*p* = 0.106) at 28 days. However, the HFNC-F group had a lower rate of ECMO at 28 days than the MV group (*p* = 0.021). Even after adjustment for covariables, the only association between the HFNC therapy and the lower likelihood of ECMO at 28 days (adjusted hazard ratio, 0.30; 95% confidence interval, 0.11–0.83; *p* = 0.045) were observed (Table 3). A detailed description of the multivariate Cox regression models for secondary outcomes was provided in supplemental Tables 3–8. There were no significant differences between the two groups concerning the length of the mechanical ventilator (13.00 days [7.00–32.00] in the MV group and 12.0 days [6.00–25.00] in the HFNC-F group, *p* = 0.849), length of stay in ICU (14.00 days [8.00–35.00] in the MV group and 16.0 days [8.00–34.00] in the HFNC-F group, *p* = 0.762), or length of hospital stay (27.00 days [16.00–95.00] in the MV group and 31.0 days [17.00–46.00] in the HFNC-F group, *p* = 0.320). The results of outcomes according to the prespecified subgroups and propensity-score matched cohort were shown in supplemental Tables 9–15 and Figs. 1 and 2.

**Table 3:** Secondary outcomes

Outcomes	MV (*n* = 51)	HFNC-F (*n* = 107)	*p*-value	Unadjusted OR (95% CI)	Adjusted OR (95% CI)
ICU mortality at day 28^a^	8 (15.7)	15 (14.0)	0.781	0.89 (0.38–2.11)	1.31 (0.46–3.71)
Successful ventilator weaning by day 28^b^	25 (49.0)	63 (58.9)	0.243	1.44 (0.91–2.29)	1.44 (0.88–2.35)
ICU discharge at day 28^c^	28 (54.9)	60 (56.1)	0.890	1.12 (0.72–1.75)	1.05 (0.66–1.70)
Prone position at day 28^d^	23 (45.1)	61 (72.6)	0.161	1.30 (0.80–2.09)	1.19 (0.73–1.95)
ECMO at day 28^e^	9 (17.6)	6 (5.6)	0.021	0.30 (0.11–0.83)	0.34 (1.12–0.98)
CRRT at day 28^f^	9 (17.6)	10 (9.3)	0.134	0.49 (0.20–1.21)	0.87 (0.29–2.63)
Duration, day					
Mechanical ventilator duration	13.00 [7.00, 32.00]	12.00 [6.00, 25.00]	0.849		
ICU length of stay	14.00 [8.00, 35.00]	16.00 [8.00, 34.00]	0.762		
Hospital length of stay	27.00 [16.00, 95.00]	31.00 [17.00, 46.00]	0.320		

Data are reported as number (percentage), median [interquartile range], or mean ± standard deviation. MV: mechanical ventilation; HFNC: high-flow nasal cannula; ECMO: extracorporeal membrane oxygenation; CRRT: continuous renal replacement therapy; ICU: intensive care unit

^a^ Multivariate model adjusted for age, comorbidities (chronic neurologic disease and chronic kidney disease), anticoagulation, use of remdesivir, and CRRT.

^b^ Multivariate model adjusted for ex-smoker, current smoker, COVID-19 vaccination, prone position, and ECMO.

^c^ Multivariate model adjusted for body mass index, chronic kidney disease, anticoagulation, prone position, and ECMO.

^d^ Multivariate model adjusted for age, body mass index, transplantation status, remdesivir, and tocilizumab

^e^ Multivariate model adjusted for age, comorbidities (chronic lung disease and chronic liver disease), and tocilizumab

^f^ Multivariate model adjusted for age, body mass index, comorbidities (chronic kidney disease and solid cancer), anticoagulation, steroid, tocilizumab, procalcitonin, and PaO2/FiO2 ratio

## Discussion

In this retrospective, single-center study, we observed that the use of HFNC before mechanical ventilation in COVID-19 patients resulted in similar changes of ventilator parameters and clinical course compared with those who immediately underwent mechanical ventilation without the use of HFNC. Furthermore, the use of HFNC was associated with a reduction in the risk of ECMO at 28 days even though there were no significant associations between the use of HFNC and clinical outcomes in mechanically ventilated COVID-19 patients. In all cases of respiratory failure, the failure of HFNC may not necessarily worsen physiological parameters and clinical outcomes.

Although several studies have analyzed the benefits of HFNC in COVID-19 patients with ARDS, most have focused on the influence of HFNC on reducing the risk of intubation. A previous study reported a decreased risk of intubation with HFNC compared with standard oxygen [10] A randomized study showed a decreased risk of intubation with HFNC compared to standard oxygen, while another study reported no difference in intubation rates between HFNC and standard oxygen [12, 13]. Recently, a randomized clinical trial conducted in France reported that the intubation rate was significantly lower with HFNC than with standard oxygen, without superiority to 28-day mortality [9]. However, the effect of HFNC failure before mechanical ventilation in patients with COVID-19 remains unknown. Although there was a difference in the PaO_2_/FiO_2_ ratio at the time of hospital admission between the two groups in our study, no difference in mechanical ventilation parameters was observed at day 1 of mechanical ventilation. In addition, patients who underwent mechanical ventilation after the failure of HFNC had a similar prognosis, including ICU mortality on day 28, to those who received invasive ventilation immediately. Thus, failure of HFNC therapy may not have a significant impact on the prognosis of COVID-19 patients with ARDS.

Two phenotypes of COVID-19-related ARDS have been proposed based on the severity of the infection, ventilator responsiveness of the patients to hypoxemia, and time interval from the onset of the disease [14]. One of the phenotypes is type-L, which may be considered as the early stage of COVID-19-related ARDS, presenting with only sub-pleural ground-glass densities on computed tomography scan, high compliance, low ventilation-to-perfusion ratio, and low lung recruitability [15]. In contrast, type H may have the key features of low compliance, high right-to-left shunt, and high lung recruitability. The transition from type L to type H may be induced by interstitial lung edema due to negative inspiratory intrathoracic pressure [16]. In patients with type-L, dyspnea leads to this phenomenon according to SILI [17]. In our study, no deterioration in lung physiology after the failure of HFNC was observed, and the use of HFNC before mechanical ventilation was even associated with a reduction in the probability of ECMO. We observed that median values of PaCO2 between initial and worst values in the HFNC-F group were similar, even though median values of FiO2 increased from the start of HFNC to the period before mechanical ventilation. Considering these findings, thus, we considered that the use of HFNC in type L patients with dyspnea may attenuate the transition to type H by decreasing SILI-induced interstitial pulmonary edema. Higher dynamic compliance on day3 in the HFNC group than that in the MV group may support our hypothesis. In contrast, several complications due to early endotracheal intubation, including aspiration of gastric contents, disruption of the natural airway defense, sedatives induced hemodynamic instability, and increased risk of VILI might have decreased dynamic compliance on day3 and increased the rate of ECMO in the MV group [18].

Mechanical ventilation often increases the risk of kidney injury [19]. Positive pressure ventilation leads to damage to the alveolar-capillary membrane, resulting in the release of proinflammatory cytokines [20]. This propagation of inflammatory cascades induces ventilator-induced kidney injury (VIKI) [21]. In our study, there was no difference in the CRRT rate according to the use of HFNC. The similarity of parameters at the initiation of mechanical ventilation as well as the same duration of mechanical ventilation between the two groups may provoke a similar impact of VIKI.

This study has several limitations that should be considered when interpreting the results. First, this was a retrospective study conducted at a single center; a study bias may have influenced the results of this study. The imbalances in baseline characteristics between the two groups may bias the study interpretation, although we adjusted for outcomes. Second, the actual effects of HFNC on SILI are unknown because there is no objective indicator of SILI. Third, the intubation in HFNC-F group was done by intensivist’s decision rather than the objective criteria such as ROX index though COVID-19 patients were managed by the dedicated ICU team. Finally, chest tomography was not conducted at admission to evaluate the type of COVID-19-related ARDS in all patients; however, chest X-rays in most patients presented similar findings, such as diffuse ground glass opacity and focal consolidation in both lungs.

## Conclusions

In conclusion, the use of HFNC before mechanical ventilation in COVID-19 patients had similar clinical outcomes compared with those with immediately mechanical ventilation in a retrospective analysis of HFNC in 158 patients with COVID-19. The failure of HFNC before mechanical ventilation may not worsen lung physiology and clinical outcomes in the case of respiratory failure with low elastance, as seen in COVID-19.

### Electronic supplementary material

Below is the link to the electronic supplementary material.

Supplementary Material 1

## Data Availability

The datasets used and/or analysed during the current study are available from the corresponding author on reasonable request.
