# Supplementary material for: Prognosis of mechanically ventilated patients with COVID-19 after failure of high-flow nasal cannula: a retrospective cohort study
Source: Respir Res. 2024 Mar 1;25:109. doi: 10.1186/s12931-024-02671-y (PMC10905875; doi:10.1186/s12931-024-02671-y)
Supplement: Supplementary file 1 — Supplementary Material 1 [file 12931_2024_2671_MOESM1_ESM.docx]

**Supplemental Table 1** Parameters of high-flow nasal cannula

|  | HFNC (n = 107) |
| --- | --- |
| High-flow nasal cannula |  |
| Total duration, days | 1.00 [1.00, 3.00] |
| Interval until mechanical ventilation, day | 1.00 [1.00, 3.00] |
| SOFA score at initiation | 3.00 [2.00, 4.00] |
| Initial values |  |
| FiO2, % | 75.00 [60.00, 95.00] |
| Flow, L/min | 50.00 [40.00, 50.00] |
| Respiratory rate, /min | 24.0 [20.50-29.50] |
| PaCO2, mmHg | 32.0 [28.6-34.7] |
| ROX index | 4.13 [3.45, 5.68] |
| Worst values |  |
| FiO2, % | 90.00 [80.00, 100.00] |
| Flow, L/min | 50.00 [45.00, 50.00] |
| PaCO2, mmHg | 31.8 [28.9-35.6] |
| ROX index | 3.32 [2.86, 3.88] |

Data are reported as median [interquartile range]. HFNC; high-flow nasal cannula, SOFA; sequential organ failure assessment, PaCO2; arterial carbon dioxide partial pressure, FiO2; fractional inspired oxygen, ROX; respiratory rate oxygenation.

**Supplemental Table 2** Profiles according to the type of respiratory support after mechanical ventilation

|  | MV (n = 51) | HFNC-F (n = 107) | *p*-value |
| --- | --- | --- | --- |
| Prone position, n (%) | 25 (49.0) | 64 (59.80) | 0.201 |
| Time interval from MV to Prone, day | 1.00 [0.0-1.00] | 2.00 [1.00-5.00] | 0.027 |
| Duration, day | 3.00 [2.00-9.50] | 3.00 [1.00-7.00] | 0.811 |
| Response for prone position | 18 (72.00) | 46 (71.90) | 0.991 |
| ECMO | 9 (17.6) | 7 (6.5) | 0.031 |
| Time interval from MV to ECMO, day | 0.0 [0.0-5.50] | 3.00 [2.00-29.00] | 0.031 |
| Duration, day | 16.0 [9.00-56.00] | 24.00 [13.00-39.00] | 0.536 |
| Prone to ECMO | 4 (7.84) | 5 (4.67) | 0.358 |
| Time interval from Prone to ECMO, day | 9.00 [3.50-26.50] | 1.0 [0.5-21.0] | 0.703 |
| Response for prone position | 4 (100.0) | 4 (80.0) | 1.000 |

Data are reported as median [interquartile range] or number (percentage). HFNC; high-flow nasal cannula, MV; mechanical ventilation, ECMO; extracorporeal membrane oxygenation.

| **Supplemental Table 3** Cox proportional hazards model of factors associated with ICU-mortality at 28 days | | | | | | | |
| --- | --- | --- | --- | --- | --- | --- | --- |
|  | **Univariable analysis** | | |  | **Multivariable analysis** | | |
| **Variable** | **OR** | **95% CI** | ***p-*value** |  | **OR** | **95% CI** | ***p-*value** |
| Age, yr | 1.033 | 0.997-1.069 | 0.070 |  | 1.026 | 0.991-1.063 | 0.147 |
| Sex, female | 1.754 | 0.769-4.003 | 0.182 |  |  |  |  |
| Body mass index, kg/m^2^ | 0.944 | 0.848-1.052 | 0.296 |  |  |  |  |
| Smoking |  |  |  |  |  |  |  |
| Ex-smoker | 1.331 | 0.491-3.612 | 0.574 |  |  |  |  |
| Current smoker | 0.329 | 0.044-2.473 | 0.280 |  |  |  |  |
| Comorbidities |  |  |  |  |  |  |  |
| Hypertension | 1.123 | 0.493-2.562 | 0.782 |  |  |  |  |
| Diabetes mellitus | 0.811 | 0.320-2.058 | 0.660 |  |  |  |  |
| Cardiovascular disease | 1.208 | 0.447-3.260 | 0.710 |  |  |  |  |
| Chronic lung disease | 0.556 | 0.075-4.129 | 0.566 |  |  |  |  |
| Chronic neurologic disease | 4.099 | 1.678-10.016 | 0.002 |  | 3.275 | 1.309-8.196 | 0.011 |
| Chronic kidney disease | 2.809 | 0.831-9.495 | 0.097 |  | 0.856 | 0.204-3.599 | 0.832 |
| Chronic liver disease | 0.045 | 0.000-65.258 | 0.403 |  |  |  |  |
| Immunocompromised | 1.218 | 0.285-5.202 | 0.790 |  |  |  |  |
| Transplantation status | 0.900 | 0.121-6.681 | 0.918 |  |  |  |  |
| Connective tissue disease | 0.717 | 0.097-5.329 | 0.745 |  |  |  |  |
| Hematologic malignancy | 1.788 | 0.240-13.306 | 0.570 |  |  |  |  |
| Solid cancer | 1.423 | 0.333-6.077 | 0.634 |  |  |  |  |
| COVID-19 |  |  |  |  |  |  |  |
| Vaccination | 1.027 | 0.381-2.771 | 0.958 |  |  |  |  |
| Community vs. Hospital | 1.478 | 0.346-6.309 | 0.598 |  |  |  |  |
| Anticoagulation | 0.141 | 0.046-0.428 | 0.001 |  | 0.149 | 0.029-0.757 | 0.022 |
| Remdesivir | 0.480 | 0.203-1.135 | 0.095 |  | 0.618 | 0.255-1.496 | 0.286 |
| Dexamethasone ≥ 6mg/day | 20.305 | 0.00-6.734 | 0.890 |  |  |  |  |
| Tocilizumab | 1.451 | 0.639-3.296 | 0.373 |  |  |  |  |
| Baricitinib | 1.927 | 0.451-8.239 | 0.376 |  |  |  |  |
| Others | 0.335 | 0.045-2.489 | 0.285 |  |  |  |  |
| PaO2/FiO2 ratio, mmHg | 0.768 | 0.145-4.073 | 0.757 |  |  |  |  |
| PaO2/FiO2 ratio before MV | 0.997 | 0.990-1.005 | 0.482 |  |  |  |  |
| Other intervention |  |  |  |  |  |  |  |
| CRRT | 2.333 | 0.959-5.677 | 0.062 |  | 1.210 | 0.381-3.847 | 0.746 |
| Prone | 0.872 | 0.367-2.072 | 0.757 |  |  |  |  |
| ECMO | 0.511 | 0.119-2.189 | 0.366 |  |  |  |  |
| Lung Transplantation | 0.042 | 0.00-21.834 | 0.321 |  |  |  |  |
| High-flow nasal cannula | 0.896 | 0.380-2.116 | 0.803 |  | 1.307 | 0.460-3.715 | 0.616 |

ICU; intensive care unit, OR; odd ratio, CI; confidence interval, COVID-19; coronavirus disease 2019, PaO2; arterial oxygen partial pressure, FiO2; fractional inspired oxygen, MV; mechanical ventilation, CRRT; continuous renal replacement therapy, ECMO; extracorporeal membrane oxygenation, HFNC; high-flow nasal cannula.

| **Supplemental Table 4** Cox proportional hazards model of factors associated with successful ventilator weaning by 28 days | | | | | | | |
| --- | --- | --- | --- | --- | --- | --- | --- |
|  | **Univariable analysis** | | |  | **Multivariable analysis** | | |
| **Variable** | **OR** | **95% CI** | ***p-*value** |  | **OR** | **95% CI** | ***p-*value** |
| Age, yr | 0.996 | 0.981-1.010 | 0.555 |  |  |  |  |
| Sex, female | 1.134 | 0.744-1.727 | 0.559 |  |  |  |  |
| Body mass index, kg/m^2^ | 1.028 | 0.980-1.079 | 0.257 |  |  |  |  |
| Smoking |  |  |  |  |  |  |  |
| Ex-smoker | 1.633 | 0.925-2.883 | 0.091 |  | 1.490 | 0.828-2.683 | 0.184 |
| Current smoker | 0.927 | 0.509-1.689 | 0.804 |  | 1.516 | 0.805-2.854 | 0.197 |
| Comorbidities |  |  |  |  |  |  |  |
| Hypertension | 1.089 | 0.716-1.655 | 0.690 |  |  |  |  |
| Diabetes mellitus | 0.931 | 0.594-1.458 | 0.754 |  |  |  |  |
| Cardiovascular disease | 0.662 | 0.331-1.321 | 0.242 |  |  |  |  |
| Chronic lung disease | 1.093 | 0.476-2.511 | 0.833 |  |  |  |  |
| Chronic neurologic disease | 0.797 | 0.323-1.966 | 0.622 |  |  |  |  |
| Chronic kidney disease | 1.123 | 0.455-2.774 | 0.801 |  |  |  |  |
| Chronic liver disease | 0.874 | 0.438-1.743 | 0.702 |  |  |  |  |
| Immunocompromised | 0.494 | 0.156-1.564 | 0.231 |  |  |  |  |
| Transplantation status | 0.899 | 0.284-2.844 | 0.856 |  |  |  |  |
| Connective tissue disease | 0.301 | 0.042-2.167 | 0.233 |  |  |  |  |
| Hematologic malignancy | 0.855 | 0.210-3.475 | 0.826 |  |  |  |  |
| Solid cancer | 1.549 | 0.626-3.835 | 0.344 |  |  |  |  |
| COVID-19 |  |  |  |  |  |  |  |
| Vaccination | 1.664 | 1.001-2.767 | 0.050 |  | 1.334 | 0.795-2.240 | 0.275 |
| Community vs. Hospital | 0.589 | 0.186-1.865 | 0.368 |  |  |  |  |
| Anticoagulation | 1.101 | 0.403-3.004 | 0.852 |  |  |  |  |
| Remdesivir | 1.525 | 0.828-2.807 | 0.176 |  |  |  |  |
| Dexamethasone ≥ 6mg/day | 0.549 | 0.076-3.974 | 0.552 |  |  |  |  |
| Tocilizumab | 1.318 | 0.865-2.007 | 0.199 |  |  |  |  |
| Baricitinib | 1.244 | 0.574-2.696 | 0.580 |  |  |  |  |
| Others | 0.689 | 0.333-1.428 | 0.317 |  |  |  |  |
| PaO2/FiO2 ratio, mmHg | 1.363 | 0.619-3.000 | 0.442 |  |  |  |  |
| Other intervention |  |  |  |  |  |  |  |
| CRRT | 0.709 | 0.287-1.754 | 0.457 |  |  |  |  |
| Prone | 0.498 | 0.325-0.764 | 0.001 |  | 0.417 | 0.268-0.649 | <0.001 |
| ECMO | 0.037 | 0.003-0.462 | 0.011 |  | 0.000 | 0.000-4.193 | 0.964 |
| Lung Transplantation | 0.042 | 0.001-1.235 | 0.066 |  |  |  |  |
| High-flow nasal cannula | 1.506 | 0.947-2.396 | 0.084 |  | 1.436 | 0.877-2.354 | 0.151 |

OR; odd ratio, CI; confidence interval, COVID-19; coronavirus disease 2019, PaO2; arterial oxygen partial pressure, FiO2; fractional inspired oxygen, CRRT; continuous renal replacement therapy, ECMO; extracorporeal membrane oxygenation, HFNC; high-flow nasal cannula.

| **Supplemental Table 5** Cox proportional hazards model of factors associated with ICU discharge at 28 days | | | | | | | |
| --- | --- | --- | --- | --- | --- | --- | --- |
|  | **Univariable analysis** | | |  | **Multivariable analysis** | | |
| **Variable** | **OR** | **95% CI** | ***p-*value** |  | **OR** | **95% CI** | ***p-*value** |
| Age, yr | 0.989 | 0.975-1.003 | 0.134 |  |  |  |  |
| Sex, female | 0.967 | 0.633-1.477 | 0.878 |  |  |  |  |
| Body mass index, kg/m^2^ | 1.041 | 0.993-1.092 | 0.098 |  | 1.051 | 1.004-1.101 | 0.034 |
| Smoking |  |  |  |  |  |  |  |
| Ex-smoker | 1.482 | 0.839-2.618 | 0.176 |  |  |  |  |
| Current smoker | 1.231 | 0.698-2.173 | 0.473 |  |  |  |  |
| Comorbidities |  |  |  |  |  |  |  |
| Hypertension | 1.075 | 0.707-1.634 | 0.736 |  |  |  |  |
| Diabetes mellitus | 0.865 | 0.550-1.361 | 0.531 |  |  |  |  |
| Cardiovascular disease | 0.692 | 0.358-1.339 | 0.275 |  |  |  |  |
| Chronic lung disease | 0.892 | 0.389-2.045 | 0.787 |  |  |  |  |
| Chronic neurologic disease | 1.127 | 0.492-2.584 | 0.778 |  |  |  |  |
| Chronic kidney disease | 2.344 | 1.008-5.451 | 0.048 |  | 1.884 | 0.676-5.251 | 0.226 |
| Chronic liver disease | 0.923 | 0.463-1.841 | 0.820 |  |  |  |  |
| Immunocompromised | 1.201 | 0.487-2.963 | 0.691 |  |  |  |  |
| Transplantation status | 1.575 | 0.496-4.999 | 0.441 |  |  |  |  |
| Connective tissue disease | 0.605 | 0.149-2.457 | 0.482 |  |  |  |  |
| Hematologic malignancy | 1.923 | 0.703-5.258 | 0.203 |  |  |  |  |
| Solid cancer | 1.381 | 0.602-3.166 | 0.446 |  |  |  |  |
| COVID-19 |  |  |  |  |  |  |  |
| Vaccination | 1.256 | 0.730-2.160 | 0.411 |  |  |  |  |
| Community vs. Hospital | 1.010 | 0.441-2.315 | 0.981 |  |  |  |  |
| Anticoagulation | 0.362 | 0.165-0.793 | 0.011 |  | 0.649 | 0.248-1.699 | 0.378 |
| Remdesivir | 1.441 | 0.765-2.714 | 0.258 |  |  |  |  |
| Dexamethasone ≥ 6mg/day | 0.395 | 0.054-2.876 | 0.359 |  |  |  |  |
| Tocilizumab | 1.192 | 0.784-1.812 | 0.411 |  |  |  |  |
| Baricitinib | 1.382 | 0.638-2.996 | 0.412 |  |  |  |  |
| Others | 0.649 | 0.313-1.345 | 0.245 |  |  |  |  |
| PaO2/FiO2 ratio, mmHg | 1.756 | 0.797-3.868 | 0.163 |  |  |  |  |
| Other intervention |  |  |  |  |  |  |  |
| CRRT | 0.742 | 0.324-1.699 | 0.480 |  |  |  |  |
| Prone | 0.474 | 0.310-0.726 | 0.001 |  | 0.430 | 0.278-0.663 | <0.001 |
| ECMO | 0.185 | 0.058-0.588 | 0.004 |  | 0.159 | 0.049-0.520 | 0.002 |
| Lung Transplantation | 0.041 | 0.002-0.894 | 0.042 |  |  |  |  |
| High-flow nasal cannula | 1.119 | 0.715-1.754 | 0.622 |  | 1.054 | 0.655-1.696 | 0.828 |

ICU; intensive care unit, OR; odd ratio, CI; confidence interval, COVID-19; coronavirus disease 2019, PaO2; arterial oxygen partial pressure, FiO2; fractional inspired oxygen, CRRT; continuous renal replacement therapy, ECMO; extracorporeal membrane oxygenation, HFNC; high-flow nasal cannula.

| **Supplemental Table 6** Cox proportional hazards model of factors associated with Prone at 28 days | | | | | | | |
| --- | --- | --- | --- | --- | --- | --- | --- |
|  | **Univariable analysis** | | |  | **Multivariable analysis** | | |
| **Variable** | **OR** | **95% CI** | ***p-*value** |  | **OR** | **95% CI** | ***p-*value** |
| Age, yr | 0.99 | 0.97-1.00 | 0.067 |  | 0.997 | 0.979-1.016 | 0.786 |
| Sex, female | 1.00 | 0.65-1.53 | 0.993 |  |  |  |  |
| Body mass index, kg/m^2^ | 1.05 | 1.01-1.10 | 0.023 |  | 1.046 | 0.990-1.106 | 0.106 |
| Smoking |  |  |  |  |  |  |  |
| Ex-smoker | 0.73 | 0.38-1.38 | 0.335 |  |  |  |  |
| Current smoker | 0.73 | 0.36-1.47 | 0.379 |  |  |  |  |
| Comorbidities |  |  |  |  |  |  |  |
| Hypertension | 0.73 | 0.47-1.12 | 0.146 |  |  |  |  |
| Diabetes mellitus | 0.86 | 0.54-1.38 | 0.537 |  |  |  |  |
| Cardiovascular disease | 0.67 | 0.35-1.30 | 0.238 |  |  |  |  |
| Chronic lung disease | 0.51 | 0.19-1.40 | 0.194 |  |  |  |  |
| Chronic neurologic disease | 0.99 | 0.48-2.06 | 0.984 |  |  |  |  |
| Chronic kidney disease | 0.65 | 0.24-1.78 | 0.407 |  |  |  |  |
| Chronic liver disease | 1.52 | 0.73-3.15 | 0.261 |  |  |  |  |
| Immunocompromised | 1.40 | 0.65-3.03 | 0.395 |  |  |  |  |
| Transplantation status | 2.28 | 0.92-5.65 | 0.075 |  | 2.908 | 1.121-7.544 | 0.028 |
| Connective tissue disease | 0.64 | 0.20-2.03 | 0.448 |  |  |  |  |
| Hematologic malignancy | 0.72 | 0.23-2.27 | 0.571 |  |  |  |  |
| Solid cancer | 1.21 | 0.53-2.79 | 0.647 |  |  |  |  |
| COVID-19 |  |  |  |  |  |  |  |
| Vaccination | 0.96 | 0.57-1.64 | 0.892 |  |  |  |  |
| Anticoagulation | 1.70 | 0.62-4.65 | 0.298 |  |  |  |  |
| Remdesivir | 1.82 | 0.97-3.44 | 0.064 |  | 1.351 | 0.689-2.649 | 0.381 |
| Tocilizumab | 1.87 | 1.21-2.89 | 0.005 |  | 1.802 | 1.137-2.856 | 0.012 |
| Baricitinib | 1.38 | 0.63-2.98 | 0.419 |  |  |  |  |
| Others | 0.88 | 0.43-1.83 | 0.735 |  |  |  |  |
| CRP | 1.00 | 0.99-1.01 | 0.402 |  |  |  |  |
| Procalcitonin | 0.97 | 0.93-1.02 | 0.222 |  |  |  |  |
| PaO2/FiO2 ratio, mmHg | 1.00 | 1.00-1.00 | 0.159 |  |  |  |  |
| High-flow nasal cannula | 1.30 | 0.80-2.09 | 0.299 |  | 1.191 | 0.727-1.950 | 0.488 |

OR; odd ratio, CI; confidence interval, COVID-19; coronavirus disease 2019, CRP; C-reactive protein, PaO2; arterial oxygen partial pressure, FiO2; fractional inspired oxygen, HFNC; high-flow nasal cannula.

| **Supplemental Table 7** Cox proportional hazards model of factors associated with ECMO at 28 days | | | | | | | |
| --- | --- | --- | --- | --- | --- | --- | --- |
|  | **Univariable analysis** | | |  | **Multivariable analysis** | | |
| **Variable** | **OR** | **95% CI** | ***p-*value** |  | **OR** | **95% CI** | ***p-*value** |
| Age, yr | 0.95 | 0.92-0.99 | 0.005 |  | 0.947 | 0.914-0.980 | 0.002 |
| Sex, female | 1.47 | 0.53-4.05 | 0.457 |  |  |  |  |
| Body mass index, kg/m^2^ | 1.05 | 0.94-1.17 | 0.381 |  |  |  |  |
| Comorbidities |  |  |  |  |  |  |  |
| Hypertension | 0.43 | 0.15-1.25 | 0.122 |  |  |  |  |
| Diabetes mellitus | 0.59 | 0.17-2.09 | 0.412 |  |  |  |  |
| Cardiovascular disease | 2.11 | 0.67-6.62 | 0.201 |  |  |  |  |
| Chronic lung disease | 3.36 | 0.95-11.90 | 0.061 |  | 6.220 | 1.520-25.451 | 0.011 |
| Chronic kidney disease | 1.10 | 0.14-8.34 | 0.929 |  |  |  |  |
| Chronic liver disease | 3.13 | 0.88-11.09 | 0.077 |  | 2.740 | 0.756-9.928 | 0.125 |
| Connective tissue disease | 1.47 | 0.19-11.17 | 0.711 |  |  |  |  |
| Solid cancer | 1.06 | 0.14-8.06 | 0.955 |  |  |  |  |
| COVID-19 |  |  |  |  |  |  |  |
| Remdesivir | 1.48 | 0.33-6.55 | 0.607 |  |  |  |  |
| Tocilizumab | 0.36 | 0.11-1.13 | 0.081 |  | 0.381 | 0.120-1.213 | 0.102 |
| Others | 0.66 | 0.09-5.02 | 0.689 |  |  |  |  |
| CRP | 0.95 | 0.89-1.02 | 0.163 |  |  |  |  |
| Procalcitonin | 0.93 | 0.76-1.13 | 0.467 |  |  |  |  |
| PaO2/FiO2 ratio, mmHg | 1.00 | 0.99-1.00 | 0.466 |  |  |  |  |
| High-flow nasal cannula | 0.30 | 0.11-0.83 | 0.019 |  | 0.336 | 0.116-0.975 | 0.045 |

ECMO; extracorporeal membrane oxygenation, OR; odd ratio, CI; confidence interval, COVID-19; coronavirus disease 2019, CRP; C-reactive protein, PaO2; arterial oxygen partial pressure, FiO2; fractional inspired oxygen, HFNC; high-flow nasal cannula.

| **Supplemental Table 8** Cox proportional hazards model of factors associated with CRRT at 28 days | | | | | | | |
| --- | --- | --- | --- | --- | --- | --- | --- |
|  | **Univariable analysis** | | |  | **Multivariable analysis** | | |
| **Variable** | **OR** | **95% CI** | ***p-*value** |  | **OR** | **95% CI** | ***p-*value** |
| Age, yr | 1.06 | 1.01-1.10 | 0.009 |  | 1.039 | 0.988-1.093 | 0.134 |
| Sex, female | 1.13 | 0.46-2.79 | 0.786 |  |  |  |  |
| Body mass index, kg/m^2^ | 0.86 | 0.76-0.97 | 0.013 |  | 0.944 | 0.836-1.065 | 0.349 |
| Smoking |  |  |  |  |  |  |  |
| Ex-smoker | 2.00 | 0.69-5.75 | 0.200 |  |  |  |  |
| Current smoker | 1.50 | 0.42-5.37 | 0.535 |  |  |  |  |
| Comorbidities |  |  |  |  |  |  |  |
| Hypertension | 1.98 | 0.75-5.21 | 0.166 |  |  |  |  |
| Diabetes mellitus | 1.82 | 0.73-4.53 | 0.197 |  |  |  |  |
| Cardiovascular disease | 1.58 | 0.52-4.75 | 0.419 |  |  |  |  |
| Chronic lung disease | 1.50 | 0.35-6.50 | 0.587 |  |  |  |  |
| Chronic neurologic disease | 1.89 | 0.55-6.47 | 0.314 |  |  |  |  |
| Chronic kidney disease | 7.37 | 2.64-20.56 | <0.001 |  | 9.496 | 2.625-34.348 | 0.001 |
| Immunocompromised | 1.87 | 0.43-8.10 | 0.402 |  |  |  |  |
| Transplantation status | 1.39 | 0.19-10.40 | 0.749 |  |  |  |  |
| Solid cancer | 2.96 | 0.86-10.14 | 0.085 |  | 1.113 | 0.233-5.309 | 0.893 |
| COVID-19 |  |  |  |  |  |  |  |
| Vaccination | 0.45 | 0.10-1.94 | 0.283 |  |  |  |  |
| Anticoagulation | 0.11 | 0.04-0.28 | <0.001 |  | 0.232 | 0.043-1.261 | 0.091 |
| Remdesivir | 0.61 | 0.22-1.69 | 0.338 |  |  |  |  |
| Dexamethasone | 0.04 | 0.00-0.32 | 0.003 |  | 0.161 | 0.007-3.587 | 0.249 |
| Tocilizumab | 0.18 | 0.05-0.62 | 0.007 |  | 0.203 | 0.052-0.789 | 0.021 |
| Others | 1.87 | 0.54-6.42 | 0.320 |  |  |  |  |
| CRP | 1.00 | 0.99-1.01 | 0.997 |  |  |  |  |
| Procalcitonin | 1.14 | 1.08-1.20 | <0.001 |  | 1.116 | 1.047-1.191 | 0.001 |
| PaO2/FiO2 ratio, mmHg | 1.00 | 1.00-1.00 | 0.002 |  | 1.006 | 0.998-1.014 | 0.159 |
| High-flow nasal cannula | 0.49 | 0.20-1.21 | 0.122 |  | 0.871 | 0.289-2.627 | 0.806 |

CRRT; continuous renal replacement therapy, OR; odd ratio, CI; confidence interval, COVID-19; coronavirus disease 2019, CRP; C-reactive protein, PaO2; arterial oxygen partial pressure, FiO2; fractional inspired oxygen, HFNC; high-flow nasal cannula.

**Supplemental Table 9** Parameters of ventilator from day 1 to day 3 after mechanical ventilation according to the pre- and post- mass vaccination

|  | Day 1 | |  | Day 2 | |  | Day 3 | |  |
| --- | --- | --- | --- | --- | --- | --- | --- | --- | --- |
|  | MV | HFNC-F | *p* | MV | HFNC-F | *p* | MV | HFNC-F | *p* |
| Pre-mass vaccination from February 2020 to October 2021 | | | | | | | | | |
| MV parameter | N = 113 | | | N = 113 | | | N = 111 | |  |
| PF ratio, mmHg | 115.8 ± 58.1 | 141.7 ± 63.0 | 0.040 | 159.6 ± 75.9 | 162.8 ± 62.8 | 0.831 | 183.2 ± 89.3 | 163.0 ± 57.6 | 0.162 |
| TV/PBW, mL/kg | 6.1 ± 1.9 | 6.3 ± 1.2 | 0.400 | 6.5 ± 1.8 | 6.5 ± 1.2 | 0.904 | 6.4 ± 1.6 | 6.8 ± 1.4 | 0.190 |
| Peak Pressure, cmH20 | 27.6 ± 5.6 | 26.7 ± 3.6 | 0.305 | 25.7 ± 5.0 | 24.9 ± 3.5 | 0.312 | 25.6 ± 5.9 | 23.7 ± 3.6 | 0.039 |
| PEEP, cmH2O | 11.9 ± 4.1 | 12.3 ± 3.3 | 0.644 | 11.1 ± 3.7 | 11.8 ± 3.0 | 0.339 | 10.9 ± 3.8 | 11.2 ± 2.4 | 0.605 |
| Dynamic C, mL/cmH20 | 24.7 [18.4, 32.2] | 26.3 [20.5, 32.6] | 0.239 | 27.3 [20.6, 39.8] | 28.8 [24.0, 36.0] | 0.397 | 30.7 [20.3, 36.3]1 | 31.1 [26.4, 38.2] | 0.106 |
| Post-mass vaccination from November 2021 to December 2021 | | | | | | | | | |
| MV parameter | N = 45 | | | N = 45 | | | N = 43 | | |
| PF ratio, mmHg | 142.2  [113.7, 184.7] | 137.9  [99.9, 208.2] | 0.862 | 185.4  [145.2, 268.6] | 174.4  [132.8, 204.4] | 0.158 | 185.0  [147.1, 222.0] | 172.2  [141.0, 238.7] | 0.802 |
| TV/PBW, mL/kg | 7.1 [6.3, 7.9] | 6.3 [5.9, 7.1] | 0.052 | 6.5 [6.0, 7.4] | 6.0 [5.8, 6.7] | 0.121 | 6.3 [5.7, 8.0] | 6.1 [5.8, 6.8] | 0.436 |
| Peak Pressure, cmH20 | 27.5  [23.5, 30.3] | 26.0  [24.0, 30.0] | 0.825 | 26.5  [20.0, 28.3] | 24.0  [22.0, 28.0] | 0.825 | 25.0  [21.0, 26.0] | 24.0  [22.0, 26.0] | 0.940 |
| PEEP, cmH2O | 13.0  [9.5, 14.0] | 13.0  [12.0, 14.0] | 0.818 | 12.0  [7.5, 12.3] | 12.0  [11.0, 14.0] | 0.029 | 10.0  [8.0, 12.0] | 11.0  [9.0, 13.0] | 0.074 |
| Dynamic C, mL/cmH20 | 26.2  [21.2, 30.3] | 27.3  [21.6, 30.3] | 0.808 | 26.4  [22.0, 29.3] | 30.4  [23.9, 35.8] | 0.219 | 25.8  [21.8, 30.0] | 29.2  [21.3, 33.9] | 0.407 |

Data are reported as number (percentage), median [interquartile range], or mean ± standard deviation. MV: mechanical ventilation; HFNC-F: high-flow nasal cannula failure; PF ratio: arterial oxygen partial pressure/fractional inspired oxygen ratio; TV/PBW: tidal volume/predicted body weight; PEEP: positive end-expiratory pressure; C: Compliance.

**Supplemental Table 10** Parameters of ventilator from day 1 to day 3 after mechanical ventilation according to the pre- and post- mass tocilizumab

|  | Day 1 | |  | Day 2 | |  | Day 3 | |  |
| --- | --- | --- | --- | --- | --- | --- | --- | --- | --- |
|  | MV | HFNC-F | *p* | MV | HFNC-F | *p* | MV | HFNC-F | *p* |
| Pre-tocilizumab from February 2020 to June 2021 | | | | | | | | | |
| MV parameter | N = 55 | | | N = 55 | | | N = 55 | |  |
| PF ratio,  mmHg | 91.1[70.0, 155.9] | 125.8  [106.8, 167.2] | 0.066 | 147.6  [81.3, 211.3] | 153.4  [133.7, 179.7] | 0.487 | 168.7  [96.7, 265.3] | 144.4  [119.5, 189.9] | 0.938 |
| TV/PBW,  mL/kg | 6.1  [5.2, 8.4] | 6.8  [6.0, 7.5] | 0.242 | 7.0  [5.9, 7.9] | 6.6  [6.2, 7.5] | 0.847 | 6.9  [6.0, 7.7] | 7.2  [6.3, 8.4] | 0.428 |
| Peak Pressure,  cmH20 | 27.0  [25.0, 30.5 | 26.0  [24.0, 27.5 | 0.137 | 25.0  [22.0, 30.0] | 25.0  [22.0, 30.0] | 0.627 | 24.5  [19.5, 28.0] | 23.0  [20.0, 26.0] | 0.399 |
| PEEP,  cmH2O | 12.0  [10.0, 14.0 | 12.0  [10.0, 12.0] | 0.488 | 12.0  [9.8, 14.0] | 12.0  [9.5, 14.0] | 0.806 | 10.0  [8.0, 14.0] | 10.0  [9.0, 12.0] | 0.708 |
| Dynamic C,  mL/cmH20 | 27.8  [18.6, 33.3] | 26.3  [22.1, 33.4] | 0.562 | 31.1  [24.7, 44.4] | 30.4  [26.5, 38.4] | 0.832 | 34.3  [20.0, 43.0] | 32.1  [27.5, 40.9] | 0.692 |
| Post-tocilizumab from July 2021 to December 2021 | | | | | | | | | |
| MV parameter | N = 103 | | | N = 103 | | | N = 99 | | |
| PF ratio,  mmHg | 135.9 ± 67.3 | 150.6 ± 72.3 | 0.302 | 192.5 ± 94.4 | 167.1 ± 67.4 | 0.154 | 165.7 ± 81.5 | 177.8 ± 61.7 | 0.300 |
| TV/PBW,  mL/kg | 6.5 ± 1.6 | 6.2 ± 1.2 | 0.202 | 6.5 ± 1.3 | 6.2 ± 1.3 | 0.226 | 6.5 ± 1.3 | 6.3 ± 1.2 | 0.451 |
| Peak Pressure,  cmH20 | 27.6 ± 5.2 | 27.7 ± 3.8 | 0.902 | 25.3 ± 4.7 | 25.4 ± 3.0 | 0.899 | 25.2 ± 4.1 | 24.3 ± 3.2 | 0.292 |
| PEEP,  cmH2O | 12.0 ± 3.8 | 12.9 ± 3.3 | 0.201 | 10.6 ± 3.5 | 12.3 ± 2.3 | 0.003 | 10.4 ± 3.4 | 11.6 ± 2.3 | 0.041 |
| Dynamic C,  mL/cmH20 | 24.9  [20.3, 30.0] | 27.0  [20.3, 30.3] | 0.552 | 25.6  [20.6, 30.5] | 27.9  [22.8, 34.4] | 0.138 | 26.2  [21.7, 30.9] | 29.5  [23.5, 34.4] | 0.049 |

Data are reported as number (percentage), median [interquartile range], or mean ± standard deviation. MV: mechanical ventilation; HFNC-F: high-flow nasal cannula failure; PF ratio: arterial oxygen partial pressure/fractional inspired oxygen ratio; TV/PBW: tidal volume/predicted body weight; PEEP: positive end-expiratory pressure; C: Compliance.

**Supplemental Table 11 Subgroup analysis of secondary outcomes according to the pre- and post- mass tocilizumab**

|  | **Pre-tocilizumab (+) (n = 55)** | |  | **Post-tocilizumab (-) (n = 103)** | |  |
| --- | --- | --- | --- | --- | --- | --- |
| Outcomes | MV  (n = 14) | HFNC-F  (n = 41) | *p*-value | MV  (n = 37) | HFNC-F  (n = 66) | *p*-value |
| ICU mortality at day 28 | 3 (21.4) | 5 (12.2) | 0.405 | 5 (13.5) | 10 (15.2) | 0.821 |
| Successful ventilator weaning by day 28 | 6 (42.9) | 20 (48.8) | 0.702 | 19 (51.4) | 43 (65.2) | 0.170 |
| ICU discharge at day 28 | 7 (50.0) | 20 (48.8) | 0.937 | 21 (56.8) | 40 (60.6) | 0.703 |
| Prone position at day 28 | 6 (42.9) | 20 (48.8) | 0.702 | 17 (45.9) | 41 (62.1) | 0.112 |
| ECMO at day 28 | 3 (21.4) | 3 (7.3) | 0.165 | 6 (16.2) | 3 (4.5) | 0.067 |
| CRRT at day 28 | 2 (14.3) | 5 (12.2) | 1.000 | 7 (18.9) | 5 (7.6) | 0.112 |

Data are reported as number (percentage). MV: mechanical ventilation; HFNC: high-flow nasal cannula; ECMO: extracorporeal membrane oxygenation; CRRT: continuous renal replacement therapy; ICU: intensive care unit.

**Supplemental Table 12** Subgroup analysis of outcomes according to the use of remdesivir

|  | **Remdesivir (+) (n = 129)** | |  | **Remdesivir (-) (n = 29)** | |  |
| --- | --- | --- | --- | --- | --- | --- |
| Outcomes | MV (n = 38) | HFNC-F (n = 91) | *p*-value | MV (n= 13 ) | HFNC-F (n = 16) | *p*-value |
| Ventilator parameter at Day 3* |  | |  |  | |  |
| PF ratio, mmHg | 177.9 [149.9, 258.4] | 161.4 [122.3, 212.0] | 0.111 | 144.8 [102.1, 204.0] | 152.0 [134.5, 187.9] | 0.544 |
| Dynamic C, mL/cmH20 | 27.9 [21.8, 31.5] | 30.7 [25.3, 37.5] | 0.017 | 31.1 [20.6, 38.4] | 31.3 [26.3, 36.0] | 0.904 |
| ICU mortality at day 28 | 3 (7.9) | 12 (13.2) | 0.551 | 5 (38.5) | 3 (18.8) | 0.406 |
| Successful ventilator weaning by day 28 | 21 (55.3) | 55 (60.4) | 0.695 | 4 (30.8) | 8 (50.0) | 0.296 |
| ICU discharge at day 28 | 23 (60.5) | 54 (59.3) | 0.900 | 5 (38.5) | 6 (37.5) | 1.000 |
| Prone position at day 28 | 19 (50.0) | 54 (59.3) | 0.338 | 4 (30.8) | 7 (43.8) | 0.702 |
| ECMO at day 28 | 8 (21.1) | 5 (5.5) | 0.020 | 1 (7.7) | 1 (6.3) | 1.000 |
| CRRT at day 28 | 6 (15.8) | 8 (8.8) | 0.350 | 3 (23.1) | 2 (12.5) | 0.632 |

Data are reported as number (percentage), median [interquartile range], or mean ± standard deviation. MV: mechanical ventilation; HFNC: high-flow nasal cannula; ECMO: extracorporeal membrane oxygenation; CRRT: continuous renal replacement therapy; ICU: intensive care unit.

* The number of patients with remdesivir (+) and (-) were 127 and 27, respectively.

**Supplemental Table 13** Subgroup analysis of outcomes according to the use of tocilizumab

|  | **tocilizumab (+) (n = 77)** | |  | **tocilizumab (-) (n = 81)** | |  |
| --- | --- | --- | --- | --- | --- | --- |
| Outcomes | MV (n = 22) | HFNC-F (n = 55) | *p*-value | MV (n = 29) | HFNC-F (n = 52) | *p*-value |
| Ventilator parameter at Day 3* |  | |  |  | |  |
| PF ratio, mmHg | 169.8 [149.9, 219.5] | 159.8 [122.3, 207.2] | 0.236 | 174.8 [105.6, 282.0] | 165.8 [128.0, 209.6] | 0.598 |
| Dynamic C, mL/cmH20 | 26.7 [21.8, 31.5] | 29.2 [23.4, 35.0] | 0.255 | 29.6 [20.6, 36.3] | 32.1 [27.0, 39.6] | 0.056 |
| ICU mortality at day 28 | 4 (18.2) | 8 (14.5) | 0.734 | 4 (13.8) | 7 (13.5) | 1.000 |
| Successful ventilator weaning by day 28 | 12 (54.5) | 36 (65.5) | 0.372 | 13 (44.8) | 27 (51.9) | 0.540 |
| ICU discharge at day 28 | 12 (54.5) | 33 (60.0) | 0.661 | 16 (55.2) | 27 (51.9) | 0.779 |
| Prone position at day 28 | 13 (59.1) | 36 (65.6) | 0.600 | 10 (34.5) | 25 (48.1) | 0.236 |
| ECMO at day 28 | 1 (4.5) | 3 (5.5) | 1.000 | 8 (27.6) | 3 (5.8) | 0.014 |
| CRRT at day 28 | 1 (4.5) | 2 (3.6) | 1.000 | 8 (27.6) | 8 (15.4) | 0.186 |

Data are reported as number (percentage), median [interquartile range], or mean ± standard deviation. MV: mechanical ventilation; HFNC: high-flow nasal cannula; ECMO: extracorporeal membrane oxygenation; CRRT: continuous renal replacement therapy; ICU: intensive care unit.

* The number of patients with tocilizumab (+) and (-) were 75 and 79, respectively.

**Supplemental Table 14** Baseline characteristics of the propensity-score matched cohort

| Characteristic | MV (n = 51) | HFNC-F (n = 51) | SMD |
| --- | --- | --- | --- |
| Age, yr | 65.00 [55.00, 74.00] | 65.00 [58.00, 70.50] | 0.004 |
| Sex, female | 20 (39.2) | 22 (43.1) | 0.08 |
| Body mass index, kg/m^2^ | 25.11 [22.01, 29.16] | 25.00 [23.06, 27.19] | 0.106 |
| Smoking status |  |  | 0.847 |
| Never smoker | 31 (60.8) | 38 (74.5) |  |
| Ex-smoker | 7 (13.7) | 13 (25.5) |  |
| Current smoker | 13 (25.5) | 0 (0.0) |  |
| Comorbidities |  |  |  |
| Hypertension | 31 (60.8) | 22 (43.1) | 0.359 |
| Diabetes mellitus | 22 (43.1) | 0 (0.0) | 1.232 |
| Cardiovascular disease | 11 (21.6) | 4 (7.8) | 0.395 |
| Chronic lung disease | 5 (9.8) | 2 (3.9) | 0.234 |
| Chronic neurologic disease | 6 (11.8) | 4 (7.8) | 0.132 |
| Chronic kidney disease | 6 (11.8) | 0 (0.0) | 0.516 |
| Chronic liver disease | 4 (7.8) | 3 (5.9) | 0.078 |
| Immunocompromised status | 2 (3.9) | 4 (7.8) | 0.167 |
| Transplantation status | 1 (2.0) | 3 (5.9) | 0.203 |
| Connective tissue disease | 2 (3.9) | 4 (7.8) | 0.167 |
| Hematologic malignancy | 0 (0.0) | 2 (3.9) | 0.286 |
| Solid cancer | 3 (5.9) | 5 (9.8) | 0.146 |
| Clinical frailty score | 3.82 (1.35) | 3.33 (1.01) | 0.41 |
| Profile of COVID-19 |  |  |  |
| Vaccination | 8 (15.7) | 14 ( 27.5) | 0.289 |
| Treatment profile |  |  |  |
| Antithrombic therapy | 43 (84.3) | 51 (100.0) | 0.61 |
| Remdesivir | 38 (74.5) | 51 (100.0) | 0.827 |
| Dexamethasone ≥ 6mg/day | 50 (98.0) | 51 (100.0) | 0.2 |
| Tocilizumab | 22 (43.1) | 35 (68.6) | 0.531 |
| Baricitinib | 3 (5.9) | 3 (5.9) | <0.001 |
| Others* | 2 (3.9) | 4 (7.8) | 0.167 |
| Inflammatory marker |  |  |  |
| C-reactive protein, mg/dL | 12.33 [7.00, 24.44] | 10.29 [3.38, 17.10] | 0.312 |
| Procalcitonin, ng/mL | 0.44 [0.19, 2.29] | 0.12 [0.06, 0.18] | 0.468 |
| PaO2/FiO2 ratio, mmHg | 80.78 [53.70, 151.02] | 125.00 [86.19, 186.02] | 0.007 |

Data are reported as number (percentage) or median [interquartile range]. SMD: standardized mean difference; MV: mechanical ventilation; HFNC: high-flow nasal cannula; COVID-19: coronavirus disease 2019; PaO2: arterial oxygen partial pressure; FiO2: fractional inspired oxygen; SOFA: sequential organ failure assessment score.

***** Others included hydroxychloroquine, lopinavir/ritonavir, convalescent plasma, and immunoglobulin.

**Supplemental Table 15** Comparison of secondary outcomes in the propensity-score matched cohort

|  | Propensity-score matched cohort | |  |  |  |  |
| --- | --- | --- | --- | --- | --- | --- |
| Outcomes | MV (n = 51) | HFNC-F (n = 51) | *p*-value | HR | 95% CI | p-value |
| Ventilator parameter at Day 3 |  |  |  |  |  |  |
| PF ratio, mmHg | 171.5 [120.8, 254.2] | 147.0 [121.8, 207.0] | 0.167 |  |  |  |
| Dynamic C, mL/cmH20 | 28.4 [21.8, 32.9] | 32.7 [27.5, 38.7] | 0.006 |  |  |  |
| ICU mortality at day 28 | 8 (15.7) | 7 (13.7) | 0.780 | 0.88 | 0.32-2.43 | 0.805 |
| Successful ventilator weaning by day 28 | 25 (49.0) | 28 (54.9) | 0.552 | 1.35 | 0.79-2.32 | 0.277 |
| ICU discharge at day 28 | 28 (54.9) | 28 (54.9) | 1.000 | 1.20 | 0.71-2.03 | 0.491 |
| Prone position at day 28 | 23 (45.1) | 34 (66.7) | 0.028 | 1.66 | 0.98-2.83 | 0.061 |
| ECMO at day 28 | 9 (17.6) | 2 (3.9) | 0.025 | 0.21 | 0.05-0.96 | 0.045 |
| CRRT at day 28 | 9 (17.6) | 4 (7.8) | 0.138 | 0.41 | 0.13-1.33 | 0.136 |

Data are reported as number (percentage). MV: mechanical ventilation; HFNC: high-flow nasal cannula; ICU; intensive care unit: ECMO: extracorporeal membrane oxygenation; CRRT: continuous renal replacement therapy.


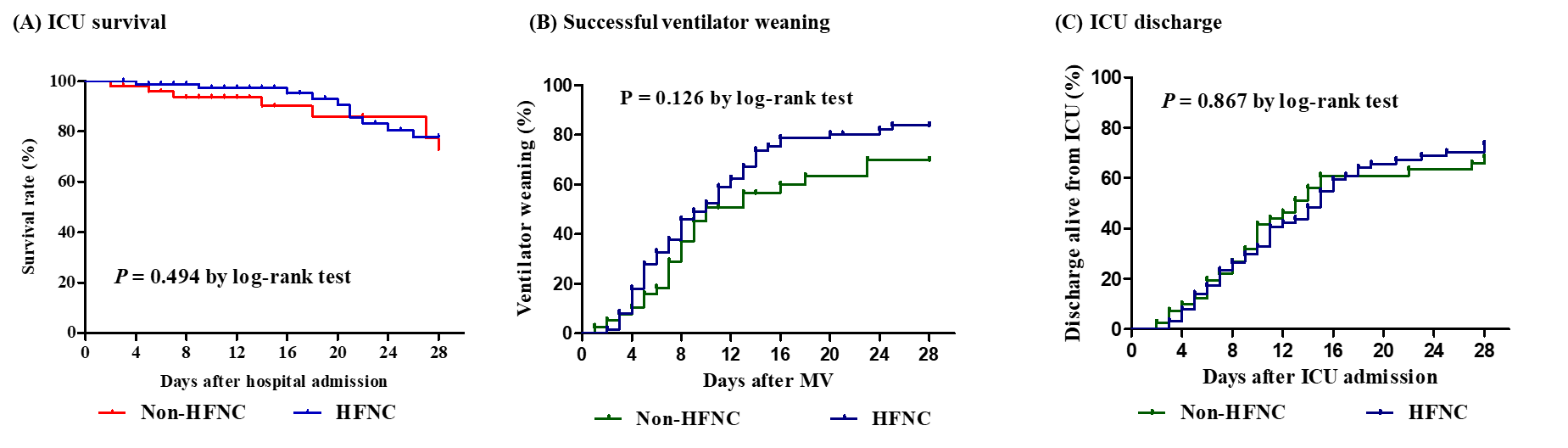


**Supplemental Figure 1.** Subgroup analyses of clinical outcomes, excluding patients intubated within 6 hours of high-flow nasal cannula exposure. (A) Intensive care unit (ICU) mortality at Day 28, (B) Successful ventilator weaning by Day 28, and (C) ICU discharge at Day 28. ICU; intensive care unit, HFNC; high-flow nasal cannula, MV; mechanical ventilation.

**
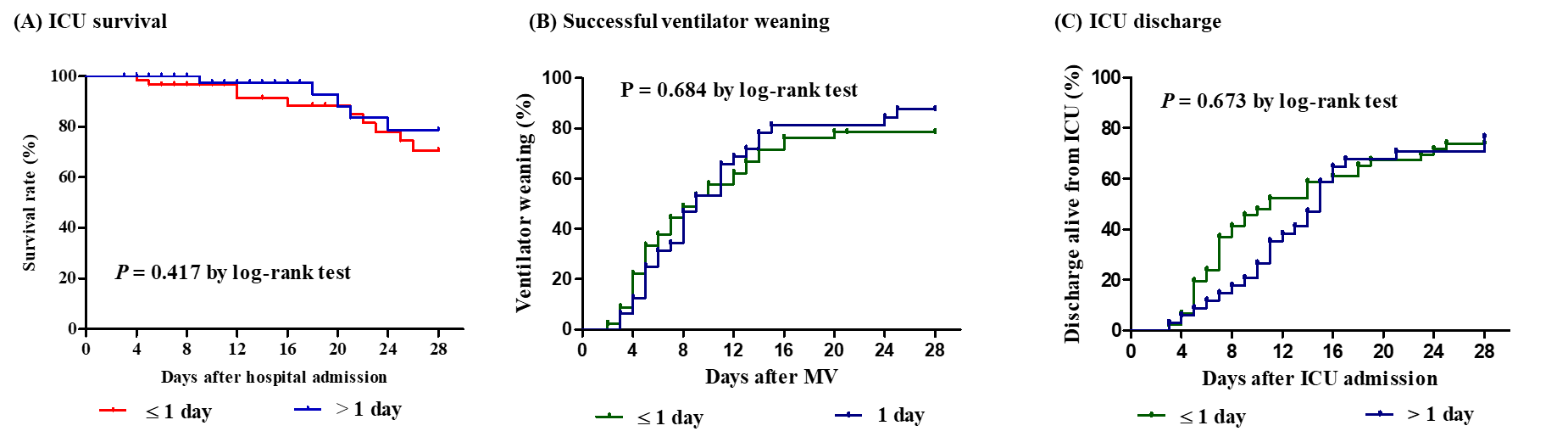
Supplemental Figure 2.** Subgroup analyses of clinical outcomes in patients with high-flow nasal cannula (HFNC) exposure according to the duration of high-flow nasal cannula. HFNC patients were divided into two groups based on the duration of HFNC ≤ 1 day or > 1 day. (A) Intensive care unit (ICU) mortality at Day 28, (B) Successful ventilator weaning by Day 28, and (C) ICU discharge at Day 28. ICU; intensive care unit, HFNC; high-flow nasal cannula, MV; mechanical ventilation.
